# Sow dimensions and loose-housed farrowing pen sizes on commercial piglet-producing farms in Sweden

**DOI:** 10.1186/s13028-024-00750-0

**Published:** 2024-07-12

**Authors:** Linda Marie Backeman Hannius, Emelie Endrésen, Helena Carlzén, Anna Wallenbeck, Rebecka Westin

**Affiliations:** 1 Department of Applied Animal Science and Welfare, Swedish University of Agricultural Sciences, Uppsala, Sweden; 2 Farm & Animal Health, Uppsala, Sweden

**Keywords:** Free farrowing, Pig, Piglet corner, Sow size, Swine

## Abstract

International interest in loose-housed farrowing is growing and there are ongoing discussions within the European Union (EU) on new legal requirements. However, there is a lack of empirical data on loose-housed farrowing pen sizes and sow dimensions in commercial production. The aim of this study was to map and describe sow size and loose-housing farrowing pen size on commercial piglet-producing farms in Sweden. The study included 146 sows and 51 pen types on 35 medium sized to large Swedish piglet-producing farms (ranging from 106 to 1300 sows in production). Sow length ranged from 129 to 238 cm (mean ± SD 191.3 ± 19.3 cm) and sow height from 74 to 133 cm (86.7 ± 7.7 cm). Floor space occupied by the sow when lying down (length x height) ranged from 1.0 to 3.2 m^2^ (1.7 ± 0.3 m^2^). Pen length ranged from 259 to 415 cm (315.1 ± 24.3 cm), pen width from 188 to 245 cm (207.0 ± 10.7 cm), total pen area from 5.7 to 8.9 m^2^ (6.5 ± 0.5 m^2^), piglet corner area from 0.5 to 1.8 m^2^ (1.1 ± 0.4 m^2^) and area available for the sow (total area - piglet corner area) from 3.9 to 6.4 m^2^ (5.4 ± 0.6 m^2^). These results show that there is substantial variation in sow, pen and piglet corner size on commercial piglet-producing farms in Sweden. This poses a risk of mismatches between sow and pen size (pens too short in relation to sow dimensions), especially for older sows. These findings are of practical significance for animal welfare and production and emphasise the importance of designing loose-housed pens adapted to future sow, litter and piglet size.

## Findings

International interest in loose-housed farrowing is growing and there are ongoing discussions within the European Union (EU) on new legal requirements in this area [1]. The European Citizens' Initiative *End the Cage Age*, initiated in 2018, is seeking a ban on farrowing crates for sows [2]. Several countries have already implemented bans on permanent farrowing crates (Norway, Sweden, Switzerland and Austria), while other countries (e.g. Netherlands and Denmark) allow farrowing crates but promote free farrowing [1]. There is a lack of empirical data on loose-housed farrowing pen size and design, and on the size range of hybrid sows in commercial production. Farmers and farm advisors claim that sows grow bigger with each parity and that sow size has increased over time (through genetic advances), posing a risk of pen size becoming insufficient. Additionally, litter size had increased, leading to a larger space requirement also for the piglets. The aim of this study was to map and describe sow dimensions and loose-housing farrowing pen sizes on commercial piglet-producing farms in Sweden.

**Fig. 1:**
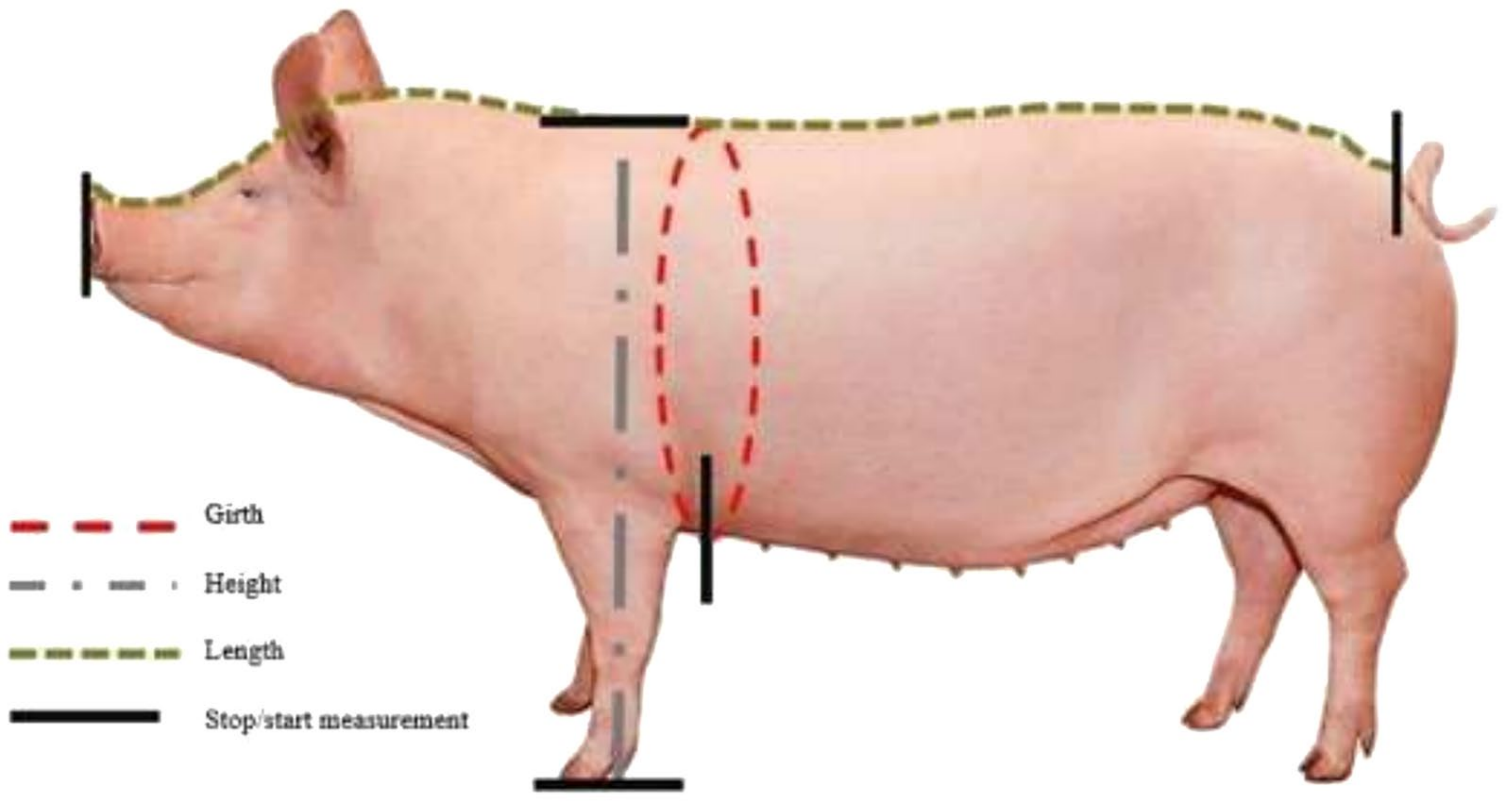
Positions at which body dimensions of the sow were measured

**Fig. 2:**
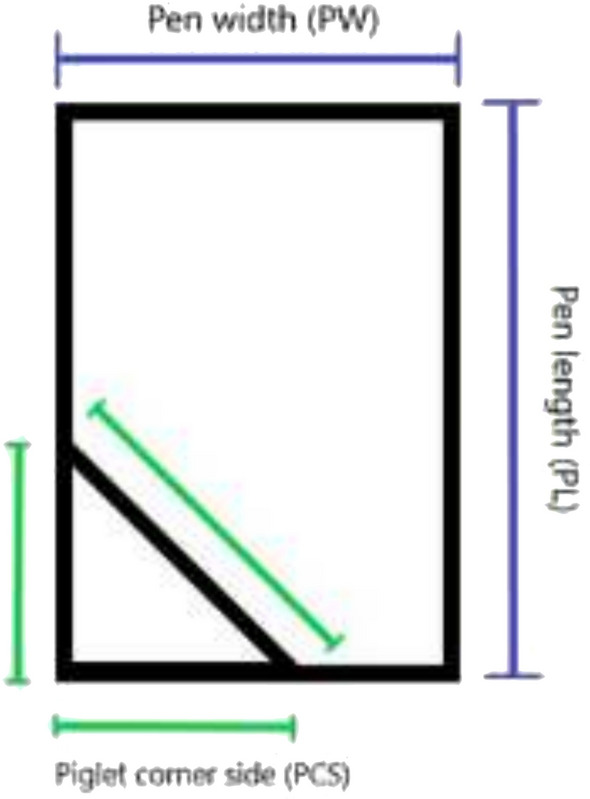
Positions at which farrowing pen and piglet corner dimensions were measured

Measurements of sow size and pen size were performed on 35 medium to large commercial piglet-producing farms in Sweden, which were visited during the period July 2022–September 2023. The farms had 106–1300 sows in production. On each farm, the body dimensions of four sows were measured (Fig. 1), aiming for the two smallest and the two largest in the batch that had farrowed most recently. The parity of each sow was also noted. In total, 146 sows ranging from parity 1 to 10 were measured (parity 1: 66 sows, parity 2: 6 sows, parity 3: 3 sows, parity 4: 6 sows, parity 5: 10 sows, parity 6: 17 sows, parity 7: 20 sows, parity 8: 10 sows, parity 9: 2 sows, parity 10: 4 sows, data on parity missing: 2 sows). Farrowing pen and piglet corner dimensions within pens were measured on each farm (Fig. 2). Fifteen of the farms had multiple farrowing houses with different pen types, so more than one pen type per farm was recorded in those cases. Twenty farms had one pen type, 14 farms had two pen types and one farm had three pen types, making 51 types of farrowing pens in total. As some of the sows were close to farrowing and could not be disturbed, piglet corner dimensions were only recorded in 35 of the 51 pen types.

**Fig. 3:**
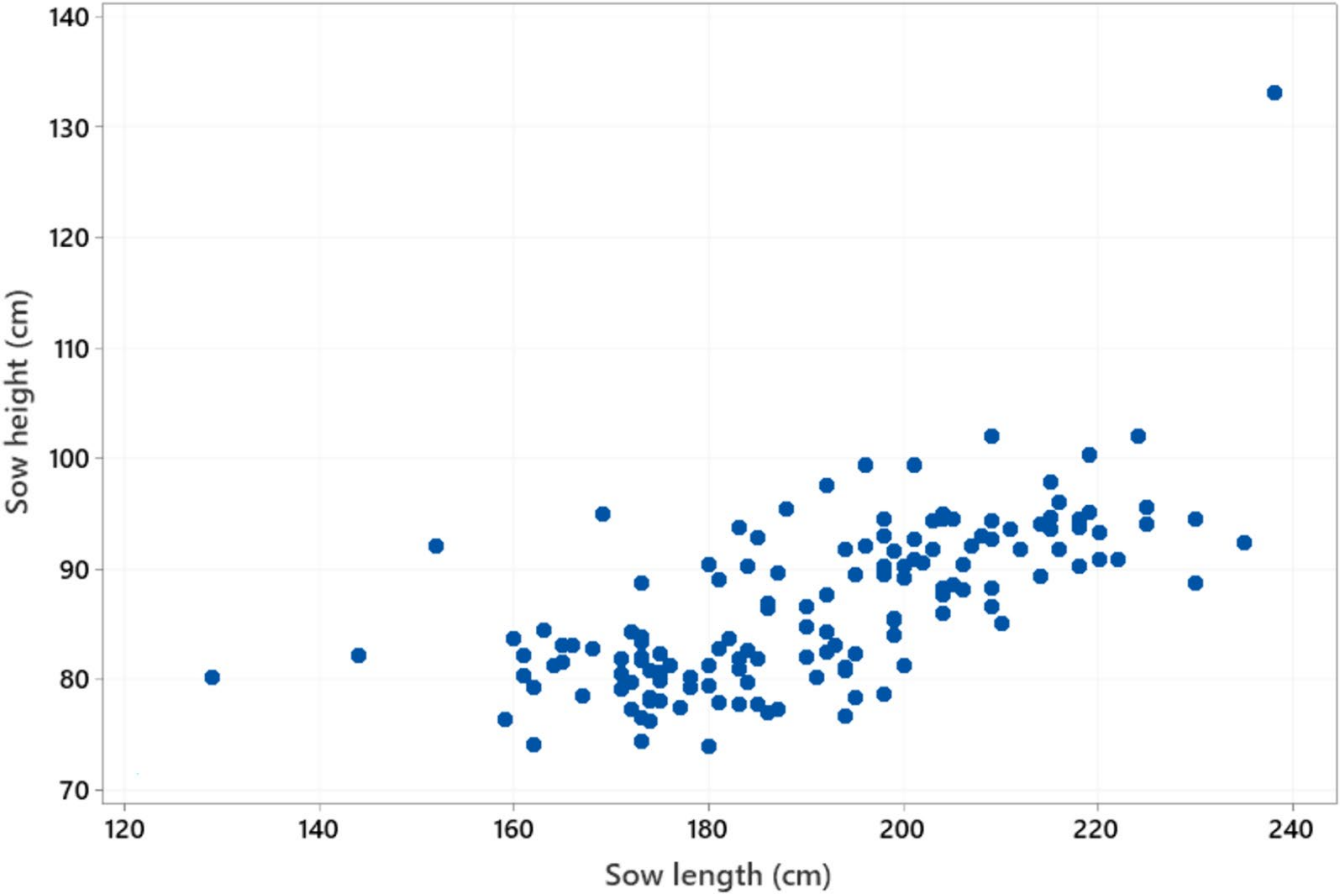
Length and height (cm) of each individual sow (n = 146 sows)

**Fig. 4:**
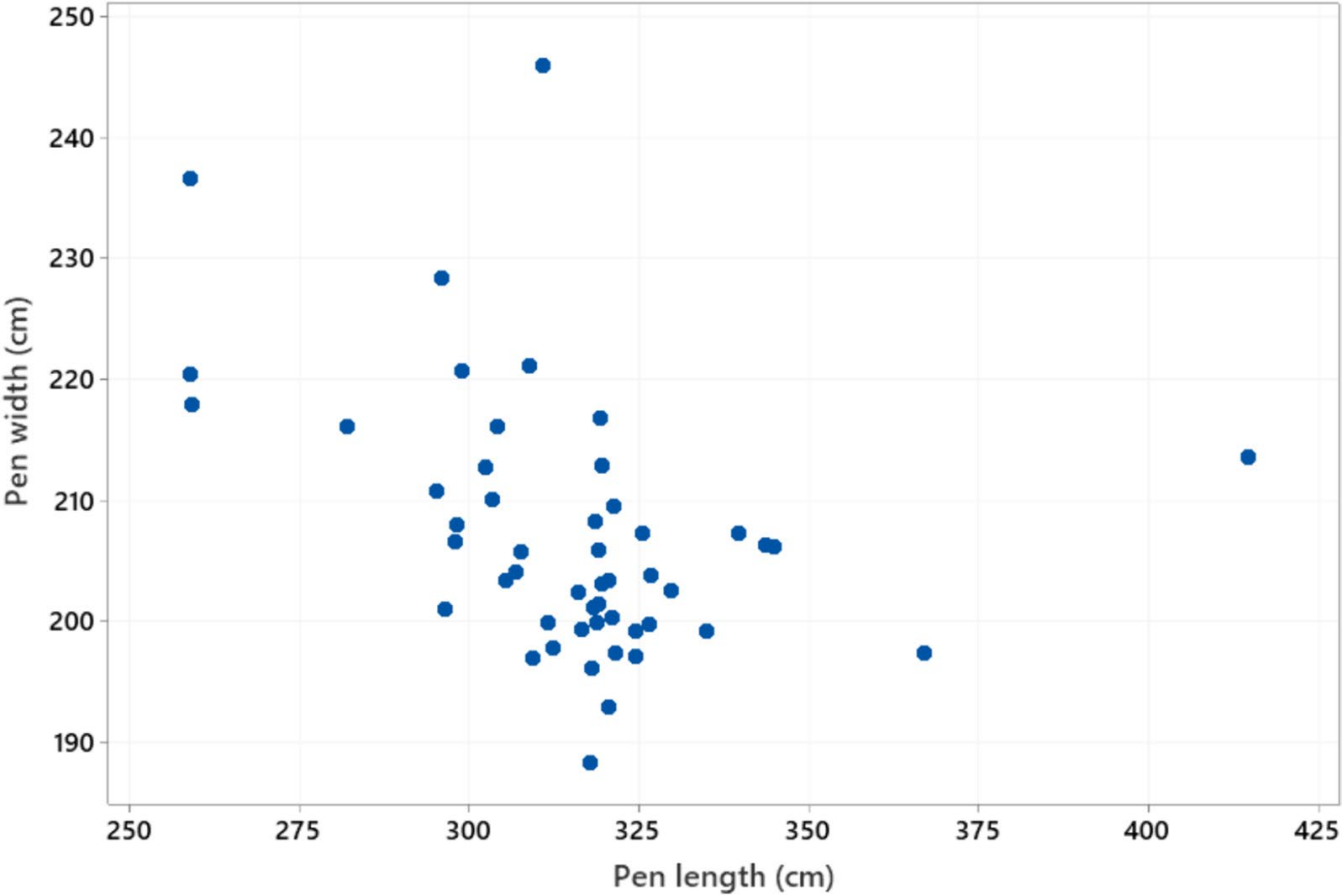
Variation in farrowing pen length and width (cm) (n = 51 pen types)

**Fig. 5:**
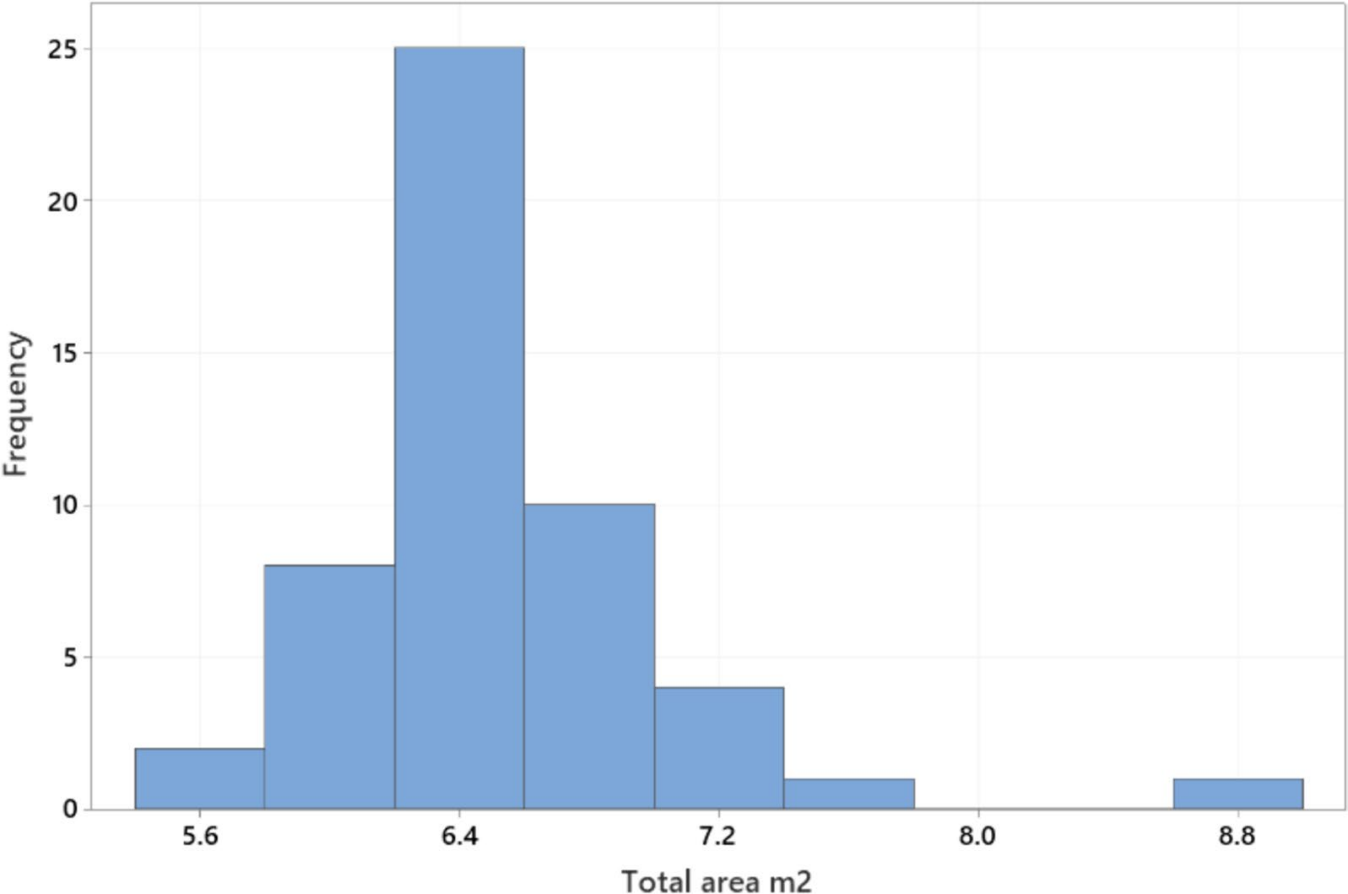
Variation in farrowing pen size (m^2^) (n = 51 pen types)

**Fig. 6:**
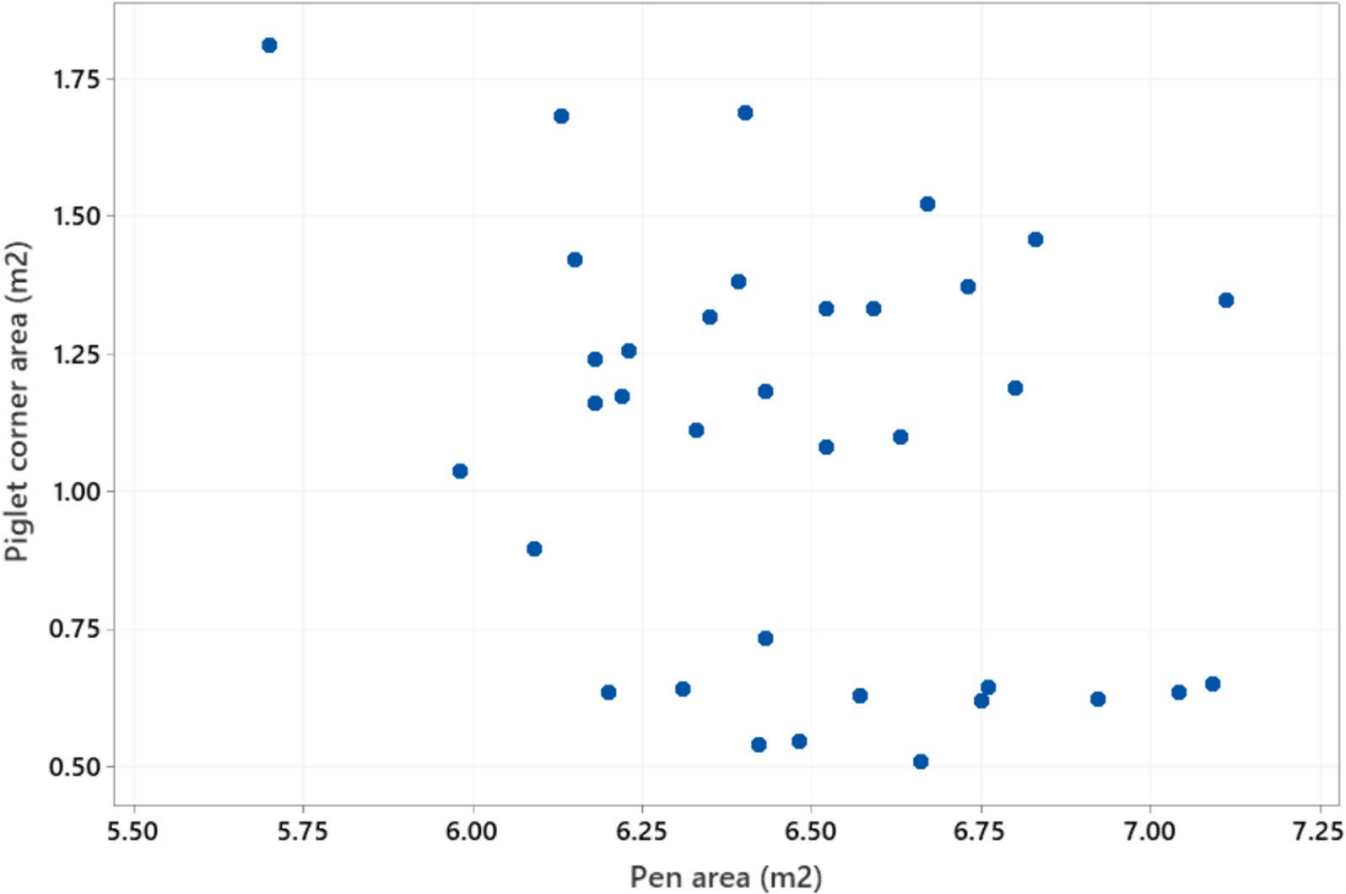
Variation in farrowing pen and piglet corner size (m^2^) (n = 35 pen types)

**Fig. 7:**
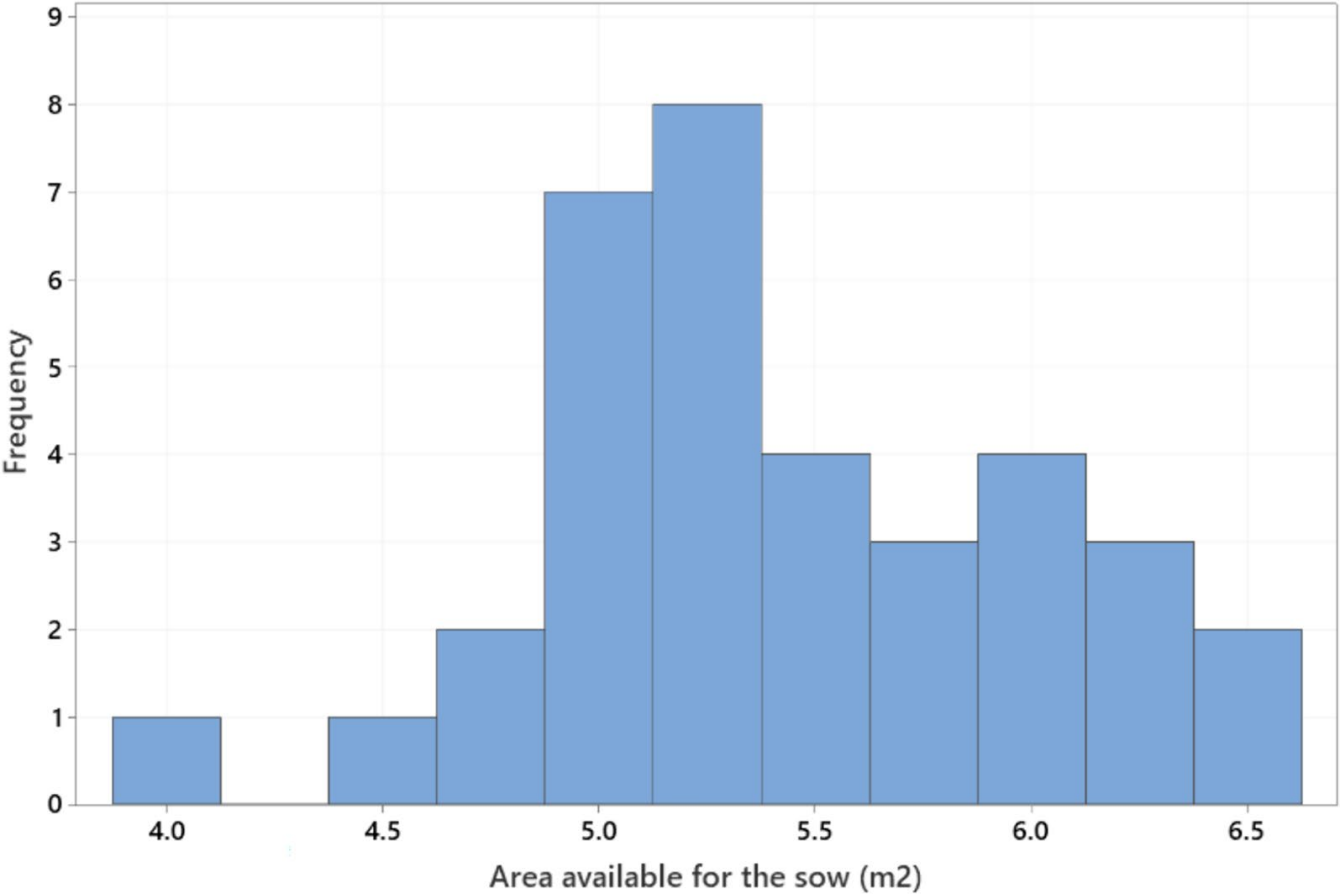
Variation in pen area available for the sow (m^2^) (n = 35 pen types)

Body length of the sows ranged from 129 to 238 cm (mean ± SD 191.3 ± 19.3) and sow height from 74 to 133 cm (86.7 ± 7.7) (Fig. 3). Floor space occupied by the sow when lying down (length x height) ranged from 1.0 to 3.2 m^2^ (1.7 ± 0.3). Girth varied from 107 to 184 cm (150.5 ± 16.2 cm). Regression analysis with a model adjusting for the fixed effect of farm (analysed with Minitab) showed that all three sow dimension variables increased significantly (P < 0.001) with parity, but that the increase flattened out at around parity 6. Pen length ranged from 259 to 415 cm (315.1 ± 24.3 cm) and pen width from 188 to 245 cm (207.0 ± 10.7 cm) (Fig. 4). Total pen area ranged from 5.7 to 8.9 m^2^ (6.5 ± 0.5 m^2^) (Fig. 5), while piglet corner area ranged from 0.5 to 1.8 m^2^ (1.1 ± 0.4 m^2^) (Fig. 6). Area available for the sow (total area - piglet corner area) ranged from 3.9 to 6.4 m^2^ (5.4 ± 0.6 m^2^) (Fig. 7).

In agreement with previous findings [3, 4], we observed that sow size increased with parity and that the increase flattened out at around parity 6. Sow length in the present study exceeded values in the most recent study (from 2018) on sow dimensions in Denmark [3]. However, those authors did not report maximum values, but rather average and 95th percentile values for the full-grown sow population (≥ parity 5). Compared with those sows, the Swedish sows in parity ≥ 5 included in this study were on average 4 cm higher and 27 cm longer, which indicates that sow size may have increased since 2018. However, the previous study estimated that sow size had not increased in Denmark from 2004 to 2018 [3]. Thus, it is possible that the difference between the two studies was instead due to differences in genetic material, as Swedish and Danish pig producers partly use different genetic lines, differences in feeding norms and/or to differences in the methods used for selection and sampling of sows.

If an increase in body size over time has actually occurred, as claimed by producers and advisors, it could be speculated that this is due to genetic changes brought about through indirect genetic selection. In other words, even though sow size is not included as a trait in the breeding goal, sow size may have increased as a correlated response to genetic selection in other traits, such as larger litters (e.g. increasing the need of longer uterus horns), greater number of teats (e.g. increasing the length of the torso) and increased growth rate. Regardless of potential effects from breeding, the results in this study confirm the claims by pig farmers and farm advisers that sow size increases with age. Thus it can be questioned whether current pen sizes are suitable for older, larger sows. To achieve the management and genetic goals for durable sows with long productive life, sow pens need to be dimensioned and designed to fit the larger high-parity sows. The large variation in sow size on commercial piglet-producing farms seen in the present study also indicates that to meet the needs of future sows, farrowing pens should be able to accommodate sows of different sizes. Possible ways to handle the variation in sow size are to have pens of different sizes within the same unit or to build pens with flexible sizes. Further studies more thoroughly quantifying the size range of hybrid sows in commercial farms, including genetic line and parity differences, are needed.

It is a complex task to design a farrowing pen that meets the requirements of both the sow and the piglets. Moreover, the needs of the sow and the piglets vary over time, i.e. they differ between farrowing, early lactation and late lactation. The continuous increase in litter size over time, the finding that older sows are larger and the potential increase in sow size related to breeding or breed differences must be considered when designing future farrowing pens. Besides absolute pen size, important factors to take into account when designing pens are the interior (e.g. protection rails and positioning of feed troughs) and the positioning and size of the functional areas of the pen (i.e. dunging, lying, feeding and piglet areas). The sow should be able to turn around in the pen and lie down with ease, and also needs sufficient space to communicate with the piglets through body language. If pen diameter is matched to sow length, the sow will have the freedom to turn around unobstructed [5, 6]. In the present study, the measured range of sow length (129–238 cm) overlapped the measured range of pen width (188–245 cm), indicating a risk of mismatches between sow and pen dimensions (pen too short in relation to sow dimensions). In the present study, the largest sows occupied up to 3 m^2^ of pen floor space when lying down and the smallest pens had less than 4 m^2^ floor space available for the sow and piglets outside the piglet corner. Taken together, these results support the European Food Safety Authority statement that a space allowance of 4 m^2^ is insufficient for sows in loose-housed farrowing pens [7].

To provide comfort and to protect piglets from being crushed by the sow, it is important that all piglets can fit into the piglet corner at the same time, during the entire nursing period until weaning. Unfortunately, literature data on the space requirement of piglets do not cover the entire nursing period up to maximum piglet size in commercial production (i.e. 5 weeks of age at weaning) [8–10]. Moreover, the current recommendations are for litter sizes of 10 pigs, while the actual number of weaned piglets has increased to on average 13–14 [11]. Based on equations used in a previous study [8], 15 pigs weighing 10 kg at weaning would occupy an area of 0.9–2.2 m^2^, depending on ambient temperature and lying position [12]. In the present study, piglet corner area ranged from 0.5 to 1.8 m^2^, which is in agreement with findings in a previous study on 33 Swedish farms that piglet corner area varies from 0.53 m^2^ to 1.72 m^2^ [13]. In combination, these results indicate a risk also of mismatches between piglet and litter size and piglet corner dimensions.

This study of sow size and loose-housing farrowing pen size on commercial piglet-producing farms in Sweden revealed substantial variations in sow, pen and piglet corner size. This variation poses a risk of mismatches between sow size and pen size of practical importance when designing loose-housed pens suitable for future sow, litter and piglet sizes.

## Data Availability

The datasets used and/or analysed in this study are available from the corresponding author on reasonable request.
